# From Amyloid Exposure to Genetic Causality: Converging hiPSC-Based Models of Alzheimer’s Disease

**DOI:** 10.3390/cells15191793

**Published:** 2026-09-30

**Authors:** Fiorella Colasuonno, Alessio Valenza, Federica Rossin, Sandra Moreno

**Affiliations:** 1 Department of Experimental Medicine, University of Rome “Tor Vergata”, 00133 Rome, Italy; fiorella.colasuonno@uniroma2.it; 2 Department of Biology, University of Rome “Tor Vergata”, 00133 Rome, Italy; 3 Department of Science, University Roma Tre, 00146 Rome, Italy; alessio.valenza@uniroma3.it; 4 LIFE Institute for Research and Health Care—Santa Lucia IRCCS, 00179 Rome, Italy; 5 Graduate School of Biomedical Sciences, University of Texas Medical Branch, Galveston, TX 77550, USA

**Keywords:** Alzheimer’s disease, stem cell therapy, iPS, CRISPR, β-amyloid, organoids, neuroinflammation, regenerative medicine, neuroglia

## Abstract

Alzheimer’s disease (AD) is a neurodegenerative disorder in which genetic susceptibility, aging, protein aggregation, and neuroglial dysfunction converge to drive progressive neuronal loss. Although animal models and conventional cell systems have provided fundamental insights into pathomechanisms, they only partially reproduce the human-specific and multifactorial nature of AD. Human induced pluripotent stem cell (hiPSC)-based models have emerged as a powerful platform to investigate disease-relevant processes within a patient-specific genetic background while enabling the generation of multiple neural cell types and multicellular systems. In this review, we present the evolution of hiPSC-based AD modeling, from reductionist paradigms based on exogenous amyloid-β exposure to CRISPR-engineered isogenic models addressing causal mutations and genetic risk variants associated with either familial or sporadic forms. We examine the contribution of three-dimensional brain organoids and neuroglial co-culture systems in recapitulating cell–cell interactions, neuroinflammatory responses, and tissue-level pathological processes. We focus on current challenges, including the limited representation of neuronal aging, variability among organoids, and the need for standardized protocols. Finally, we discuss the translational potential of hiPSC-derived systems for target identification, drug discovery, and regenerative medicine. Collectively, these advances position hiPSC-based platforms as essential complementary models for elucidating AD pathogenesis and accelerating the development of precision therapeutic strategies.

## 1. Introduction

Alzheimer’s disease (AD) is an increasingly common, age-related neurodegenerative disorder that gradually disrupts the functional organization of the brain. Although impairment of episodic memory represents its most frequent and recognizable clinical manifestation, AD is a multifaceted disorder that extends far beyond memory dysfunction. Also, many aspects of AD pathogenesis remain unresolved, particularly the mechanisms linking genetic risk, protein aggregation, aging, and dysfunction across different brain cell types.

Human post-mortem tissue is crucial for detecting disease-related molecular and cellular changes in their original anatomical environment. However, samples are typically obtained from patients at advanced stages of neurodegeneration and provide limited information about the timeline of disease progression.

Therefore, animal models, particularly genetically engineered mice, have played a central role in exploring amyloid and tau pathology, neuronal circuit dysfunction, behavioral alterations, and cell interactions within a living organism [1,2]. Nevertheless, species-specific biological differences represent an important limitation of animal models. Indeed, translation of animal research depends on the conservation of disease processes between rodents and humans, but as our understanding of the human brain and pathogenic pathways evolves, an increasing number of disparities between species arise [3]. Although rodent and human brains share major cell populations and signaling pathways, differences in macro-to-microscopic anatomy (i.a., lissencephalic vs. gyrencephalic brain, neocortical layering, hippocampal organization), gene regulation, protein function, aging mechanisms, lipid metabolism, and immune responses can influence how AD-associated processes are reproduced experimentally. Transcriptomic analyses have even revealed divergence concerning key neuroinflammatory processes [4]. Notably, AD risk genes are more abundantly expressed in brain endothelial cells in humans than in mice, and APOE4-expressing microglia in human-derived models show less efficient amyloid-β (Aβ) clearance than their APOE3 counterpart [3]. Mouse models therefore remain invaluable for testing defined mechanisms in an intact mammalian organism but are less suited to capturing the complex interactions among human genetic background, cellular identity, and aging that contribute to AD susceptibility and progression. Human induced pluripotent stem cell (hiPSC)-based models offer a complementary strategy to address these limitations. Patient-derived hiPSCs retain donor-specific genomic information and can be differentiated into virtually all neural cell types—neurons, astrocytes, microglia, oligodendrocytes, ependyma—and neurovascular cells. This approach enables direct examination of familial mutations, common susceptibility alleles, and sporadic disease backgrounds in human cells. Genome editing can also generate isogenic pairs in which relevant variants are introduced or corrected while preserving the remaining genetic background. These platforms facilitate the investigation of early, potentially reversible cellular phenotypes, the comparative effects of mutations on different cell types, and the distinction between cell-autonomous mechanisms and those arising from multicellular interactions [1,5,6]. Organoid technology and multicellular systems, allowing researchers to reproduce the complexity of specific brain regions, promise to provide invaluable tools for translating mechanistic studies into novel therapeutic approaches.

In this review, we discuss the main hiPSC-based approaches currently used to model AD, including exogenous amyloid-β exposure, CRISPR-engineered isogenic lines, and multicellular two- and three-dimensional platforms. We also consider their contribution to understanding disease mechanisms and supporting therapeutic discovery, while critically discussing the biological and technical hurdles that currently limit their translational relevance.

## 2. AD: From Clinical Complexity Towards a Biology-Based Definition

During AD progression, neuronal impairment affects multiple cognitive domains, including language, visuospatial processing, executive functions, and behavior, leading to loss of functional independence [7,8]. The sequence and rate of cognitive and functional decline vary substantially among patients. Consequently, AD should not be considered a single, uniformly evolving clinical condition, but rather a heterogeneous disorder encompassing multiple phenotypic and temporal trajectories [7,9,10].

This complexity is emphasized by the distinction between the biological development of AD and its clinical manifestation. The 2018 National Institute on Aging–Alzheimer’s Association Research Framework established a biology-based definition of AD, moving beyond dementia diagnosis. The revised 2024 diagnostic and staging criteria further develop this perspective by incorporating advances in fluid and imaging biomarkers and distinguishing the biological diagnosis of AD from the clinical staging of cognitive and functional impairment [8,11]. Even the existence of Non-Demented with Alzheimer’s Neuropathology (NDAN) individuals challenges the traditional view that AD neuropathology inevitably leads to cognitive decline [12]. Despite the presence of extensive AD-related histopathological hallmarks, these individuals remain cognitively intact, suggesting the existence of resilience mechanisms, preserving neuronal and synaptic function [13,14,15]. In addition, symptomatic patients are staged according to the severity of clinical manifestation, emphasizing that dementia is an advanced stage resulting from a much longer pathogenic process, rather than reflecting AD biological onset [8].

Within this *continuum*, the accumulation of amyloid-β peptide (Aβ) and pathological tau is accompanied by progressive alterations in synaptic integrity, neuronal connectivity, and cellular homeostasis. However, these changes should not be interpreted as a rigid and predictable cascade. Particularly in sporadic AD, amyloid pathology and tau pathology interact with aging, genetic background, and metabolic and vascular dysfunction, producing partially distinct pathological features among individuals [16]. As these processes converge, they progressively compromise synaptic function and reduce neuronal networks’ ability to adapt to pathological stress. Synaptic degeneration, which is strongly associated with cognitive decline, may therefore represent a critical point of convergence between protein pathology, altered glial responses, and subsequent neuronal loss [17]. Moreover, recent single-cell analyses of the human brain have revealed marked regional and cell-type-specific differences in vulnerability, response, and resilience to AD pathology, further supporting the view that neurodegeneration arises from the interaction of multiple cellular processes rather than from an ordered sequence of events [18].

### Neuropathological Hallmarks and Genetic Susceptibility in AD

Beyond its clinical manifestations, AD is defined by a complex neuropathological landscape involving protein aggregation, glial responses, and progressive cellular dysfunction. First described by Alois Alzheimer in 1906, the two most distinctive lesions are extracellular plaques and intracellular neurofibrillary tangles (NFT) [19]. Plaques primarily consist of Aβ peptide, produced through the sequential cleavage of amyloid precursor protein (APP) by β- and γ-secretase complexes. Among Aβ species, those with a higher propensity to aggregate form soluble oligomers and subsequently fibrillar deposits within the brain parenchyma [20]. In contrast, NFT results from the abnormal modification and accumulation of tau, a microtubule-associated protein encoded by the MAPT gene. Under physiological conditions, tau stabilizes microtubules and supports axonal transport, whereas in AD, hyperphosphorylation decreases tau’s affinity for microtubules, promoting its misfolding and aggregation. Pathological tau predominantly accumulates within neurons, forming filamentous inclusions that are closely linked to neuronal dysfunction and disease progression [21]. Although amyloid plaques and tau tangles are the defining histopathological hallmarks of AD, they constitute only a part of the broader disease process. Reactive changes in microglia are consistently observed, particularly in regions adjacent to amyloid deposits. These glial responses are not simply passive outcomes of protein accumulation; rather, they can modulate amyloid clearance, synaptic remodeling, inflammatory signaling, lipid metabolism, and neuronal homeostasis [22,23].

**Figure 1:**
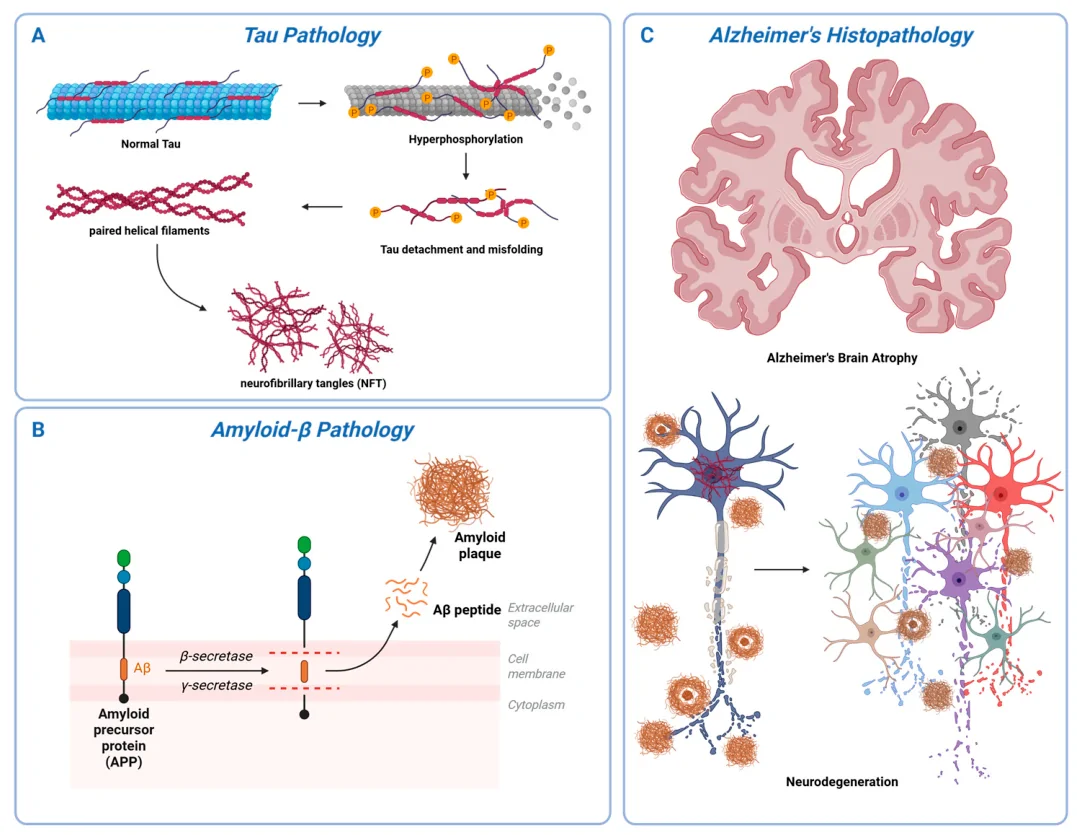
Overview of AD histopathology. (**A**) Schematic representation of tau pathology: Hyperphosphorylation leads to tau detachment from microtubules, promoting its aggregation into filaments and neurofibrillary tangles. (**B**) Amyloid-β formation: Sequential cleavage of amyloid precursor protein (APP) by β- and γ-secretases generates Aβ peptides, which accumulate in the extracellular space and form amyloid aggregates and plaques. (**C**) Histopathological features of Alzheimer’s disease, including cerebral atrophy, amyloid plaque deposition, pathological tau accumulation, neuronal loss, and altered interactions between neurons and glial cells. This figure was created with BioRender.com.

Historically, definitive diagnosis of AD required post-mortem identification of Aβ plaques and NFT. Recent advances in molecular imaging and fluid biomarkers now enable detection of core pathological changes in living individuals, supporting a biological definition of the disease and facilitating investigation across preclinical and symptomatic stages [24]. The extent and distribution of these pathological features vary significantly among patients, suggesting that their development is influenced by multiple modifying factors. Genetic background is particularly important, encompassing rare highly penetrant mutations that cause familial AD as well as common and rare variants that affect susceptibility, age at onset, cellular responses, and disease progression in sporadic cases [25] (Figure 1).

AD is typically classified based on the age at which symptoms first appear: early-onset Alzheimer’s disease (EOAD) is characterized by cognitive symptoms developing before 65 years of age, while late-onset Alzheimer’s disease (LOAD) refers to symptoms at or after this age. Both forms exhibit the core neuropathological features of amyloid-β deposition, tau aggregation, synaptic dysfunction, and progressive neurodegeneration. However, EOAD is more commonly associated with atypical, non-amnestic presentations and may be associated with a more aggressive clinical course in certain patients [9,26].

Importantly, the distinction between EOAD and LOAD is based on age at symptom onset rather than genetic etiology. Both forms may occur as familial or apparently sporadic disease, although familial aggregation is more common in EOAD. Within this group, only a minority of cases follow an autosomal-dominant inheritance pattern caused by pathogenic variants in APP, PSEN1, or PSEN2. However, most individuals with EOAD do not have a recognized causative mutation and may lack a clear family history, suggesting that early onset may result from a complex interplay between rare susceptibility variants and polygenic risk [18,27]. In addition, although LOAD is primarily sporadic and multifactorial, familiarity remains common, indicating a significant inherited component to disease [27,28].

The genetic landscape of AD includes rare variants with direct causal effects and more common alleles that influence disease susceptibility. Autosomal-dominant AD is most often linked to pathogenic variants in APP, PSEN1, and PSEN2. The APP gene encodes the precursor protein for amyloid-β peptides, whereas PSEN1 and PSEN2 encode essential components of the γ-secretase complex that mediates APP cleavage. These pathogenic variants can alter amyloid-β production or shift the relative abundance of aggregation-prone peptide species. Notably, PSEN1 variants account for the majority of genetically resolved autosomal-dominant AD cases [27,29].

The genetic architecture of LOAD is particularly complex. The APOE gene is the most significant common genetic modifier of AD risk. The APOE ε4 allele increases lifetime risk and typically leads to earlier disease onset, whereas the ε2 allele confers relative protection compared with ε3. Beyond its role in amyloid deposition and clearance, APOE also influences lipid transport, synaptic function, glial reactivity, tau-associated neurodegeneration, and vascular integrity [30,31]. Other common and rare variants have been identified in genes such as TREM2, SORL1, ABCA7, BIN1, PICALM, and CLU. These genes are involved in interconnected biological processes, including microglial immunity, lipid homeostasis, endosomal trafficking, phagocytosis, and lysosomal degradation.

Collectively, genetic evidence supports a model in which diverse molecular alterations converge on partially overlapping mechanisms, differently affecting brain cell types and resulting in age-dependent effects across individuals [27,32]. Such complexity underscores the need for human experimental models that retain patient-specific genetic information and allow the study of disease-relevant phenotypes in various brain cell types.

## 3. In Vivo and In Vitro AD Modeling

### 3.1. Transgenic Rodent AD Models

Most established AD transgenic models carry human gene variants linked to familial AD. For example, Tg2576 mice overexpress APP with the Swedish double mutation K670N/M671L, while APP/PS1 mice combine this mutation with a PSEN1 exon 9 deletion (PSEN1-ΔE9) [33]. This valuable mouse strain reproduces age-dependent changes in a relatively physiological timeline, showing morphological, ultrastructural, molecular and electrophysiological features closely resembling a human phenotype [34]. Even though relevant pathomechanisms have been dissected in this model [35,36,37], a lack of tau pathology limits its translational potential in biomedical research [2].

Conversely, the 3xTg-AD model pairs the APP Swedish and PSEN1 M146V mutations with the human MAPT P301L mutation to produce both amyloid- and tau-related pathology. Notably, P301L is a mutation associated with familial frontotemporal tauopathy rather than AD, showing that mutations from related neurodegenerative diseases are sometimes employed to replicate specific pathological features that mice do not naturally develop [2,38]. The widely used 5xFAD model includes three APP mutations—Swedish K670N/M671L, Florida I716V, and London V717I—as well as PSEN1 M146L and L286V mutations, leading to rapid and extensive cerebral amyloid accumulation. These models offer key insights into amyloid production, gliosis, synaptic dysfunction, tau accumulation, and cognitive impairment. However, their pronounced phenotypes often result from mutations and overexpression that exceed natural levels. For instance, 5xFAD mice rapidly develop amyloid plaques and inflammation but lack true neurofibrillary tangles.

Compared with conventional APP-overexpressing mouse models, TgF344-AD rats develop a broader spectrum of age-dependent AD-like alterations, including amyloid deposition, tau pathology, and cognitive impairment. The model expresses human APP carrying the Swedish mutation together with PSEN1ΔE9, although its reliance on familial AD mutations and transgenic overexpression still limits its ability to reproduce sporadic LOAD [39]. Newer knock-in and gene-edited models reduce some artifacts but still do not fully capture the age-related, polygenic, and multifactorial nature of sporadic LOAD [2].

Notwithstanding the crucial value of rodent models for mechanistic and translational studies, species-specific differences from human brain features constitute an undiscussed limitation. Notably, cross-species transcriptomic analyses have revealed substantial divergence between rodent and primate microglia, particularly in gene programs related to immune sensing, phagocytosis, complement signaling, and neurodegeneration [4]. Such differences are especially relevant to LOAD-associated genes expressed predominantly in microglia, including TREM2, CD33, CR1, and members of the MS4A family, whose sequence, regulation, or functional context may be only partially conserved between mice and humans [1].

### 3.2. In Vitro Modeling of Amyloid Pathology

In vitro AD modeling addresses the consequences of either Aβ- or p-tau-mediated neurotoxicity using different experimental settings. While tau-based models are particularly valuable for investigating microtubule destabilization, cytoskeletal abnormalities, axonal transport defects, and the progressive formation of neurofibrillary tangles, Aβ-based models remain the most common experimental platform for investigating the early molecular and cellular events associated with AD [40,41,42,43]. Therefore, the following section will focus on in vitro models of amyloid pathology, given their extensive use for evaluating candidate disease-modifying therapeutic strategies.

Aβ peptides used in in vitro models differ in sequence length, aggregation propensity, and biological activity, making the choice of isoform dependent on the experimental objective. Among the peptides generated by sequential β- and γ-secretase cleavage of APP, Aβ_1–40_ and Aβ_1–42_ are the predominant species in the human brain [41]. Aβ_1–40_ represents approximately 90% of total Aβ production and is relatively soluble, with a lower tendency to self-aggregate. In contrast, Aβ_1–42_ is less abundant but contains two additional hydrophobic amino acids at the C-terminus, which markedly increase its aggregation propensity and neurotoxicity. As a result, Aβ_1–42_ peptide rapidly forms soluble oligomers and fibrillar aggregates and is widely used to induce AD-like phenotypes in vitro. Other naturally occurring isoforms, including Aβ_1–38_, Aβ_1–37,_ Aβ_1–39_, and Aβ_1–43_, are generated by alternative γ-secretase cleavage sites. Although less extensively studied, these peptides differentially influence aggregation kinetics and disease progression. Particularly, Aβ_1–38_ is considered relatively less aggregation-prone and may even attenuate Aβ_1–42_ toxicity in some experimental settings, whereas Aβ_1–43_ displays an aggregation propensity and neurotoxicity comparable to, or even greater than, Aβ_1–42_ [40,44,45].

In recent years, exposure to exogenous soluble Aβ oligomers has become one of the most widely adopted experimental paradigms for modeling early AD-related neurotoxicity in vitro. This approach has been extensively applied across a broad range of neuronal systems, including primary rodent neurons, differentiated SH-SY5Y neuroblastoma cells, human induced pluripotent stem cell (hiPSC)-derived neurons, and, more recently, three-dimensional brain organoids. In primary cortical and hippocampal neuronal cultures, soluble Aβ oligomers induce early synaptic dysfunction, oxidative stress, mitochondrial impairment, and calcium dysregulation [46,47,48,49]. Similar neurotoxic effects, including mitochondrial dysfunction, endoplasmic reticulum stress, autophagic alterations, and apoptosis, have also been extensively documented in differentiated SH-SY5Y cells exposed to oligomeric Aβ [50,51,52,53]. More recently, hiPSC-derived neurons have provided a human-based platform for investigating Aβ oligomer toxicity, reproducing synaptic deficits, aberrant calcium signaling, and neurodegenerative phenotypes relevant to early-stage AD [5,54]. Likewise, brain organoid models have emerged as advanced multicellular systems capable of recapitulating Aβ-induced neurodegeneration, neuroinflammatory responses, oxidative stress, and protein aggregation in a three-dimensional context [55,56]. These complementary models provide increasing levels of biological complexity, ranging from highly reproducible immortalized cell lines to human neuronal cultures and multicellular tissue-like systems. Upon exposure to soluble Aβ oligomers, they consistently reproduce several key pathological features of early AD, substantially contributing to elucidating the molecular mechanisms underlying Aβ-mediated neurodegeneration.

It is important to underline that the reproducibility and experimental flexibility of exogenous Aβ paradigms have made these models particularly valuable for identifying molecular targets and evaluating candidate disease-modifying therapies in preclinical settings. Indeed, these systems also provide controlled and reproducible exposure conditions, making it possible to dissect dose-dependent and temporal effects of Aβ on synaptic function, calcium homeostasis, mitochondrial integrity, and cell survival.

Among the aforementioned models, hiPSC-derived neurons have emerged as a physiologically relevant human platform, allowing the investigation of Aβ-induced toxicity in mature neuronal populations while preserving the human genetic background. Likewise, brain organoids derived from hiPSC better recapitulate the three-dimensional architecture and cellular heterogeneity of the human brain, enabling the study of more complex pathological responses and cell–cell interactions following Aβ exposure [1,52,54,57]. Indeed, even in the absence of disease-causing mutations, the use of hiPSC-derived neurons exposed to toxic Aβ peptides offers several benefits to modeling sporadic, late-onset forms of the disease. One key advantage is human biological relevance: hiPSC-derived neurons retain the genetic background of healthy donors, providing a physiologically human context that is closer to the in vivo brain than rodent models or immortalized cell lines. This is especially relevant in AD, where subtle human-specific regulatory mechanisms may influence neuronal vulnerability to Aβ toxicity. Another major benefit is modeling sporadic AD-like stress without genetic bias. Since most AD cases are not driven by single-gene mutations, applying exogenous Aβ allows researchers to mimic a pathogenic extracellular insult while keeping the intrinsic neuronal genome unaltered. This strategy facilitates the study of Aβ-induced cellular processes, including synaptic dysfunction, mitochondrial and metabolic impairment, oxidative stress, calcium dyshomeostasis, and inflammatory signaling, thereby enabling the direct effects of extracellular Aβ to be distinguished from those secondary to familial AD mutations.

A further advantage of this technology is developmental maturation flexibility, since human hiPSC-derived neurons can be studied at different maturation stages, allowing investigation of how neuronal maturity influences susceptibility to Aβ oligomers [58]. This is relevant because aging is a major risk factor in AD, and maturity-dependent vulnerability is difficult to capture in animal models [59]. In addition, these models enable high-content functional readouts in human neurons, including electrophysiology, synaptic density measurements, transcriptomic profiling, and imaging-based assays. This supports mechanistic studies of early synaptic dysfunction, which is thought to precede overt neurodegeneration in AD. Finally, hiPSC-derived neuronal systems are highly suitable for drug screening and target validation under human-relevant conditions. Because they can be generated at scale and established from genetically distinct donors, they can also be used to assess genetic background-dependent variability in neuronal responses to Aβ toxicity, an increasingly important consideration in AD research [60].

In this context, the workflow described by Colasuonno et al. (2026) further standardized cell death detection in hiPSC-derived cortical neurons after Aβ_1–42_ exposure using TUNEL-based assays and morphological analyses, facilitating reproducible assessment of neurotoxicity. Indeed, the protocol described by the authors represents a versatile platform that can be applied not only to investigate the molecular and cellular mechanisms underlying amyloid-induced neurotoxicity but also to evaluate potential therapeutic strategies designed to prevent or reduce neuronal damage [54].

Beyond its direct effects on neurons, extracellular Aβ can elicit microglial responses that influence the progression of neuronal dysfunction. In microglia-containing cultures, Aβ exposure enables the investigation of changes in phagocytic activity, inflammatory mediator release, and complement-associated signaling, which may affect synaptic integrity and neuronal survival. These systems therefore provide an opportunity to distinguish direct amyloid-induced neurotoxicity from neuronal alterations arising through microglia-mediated mechanisms.

**Figure 2:**
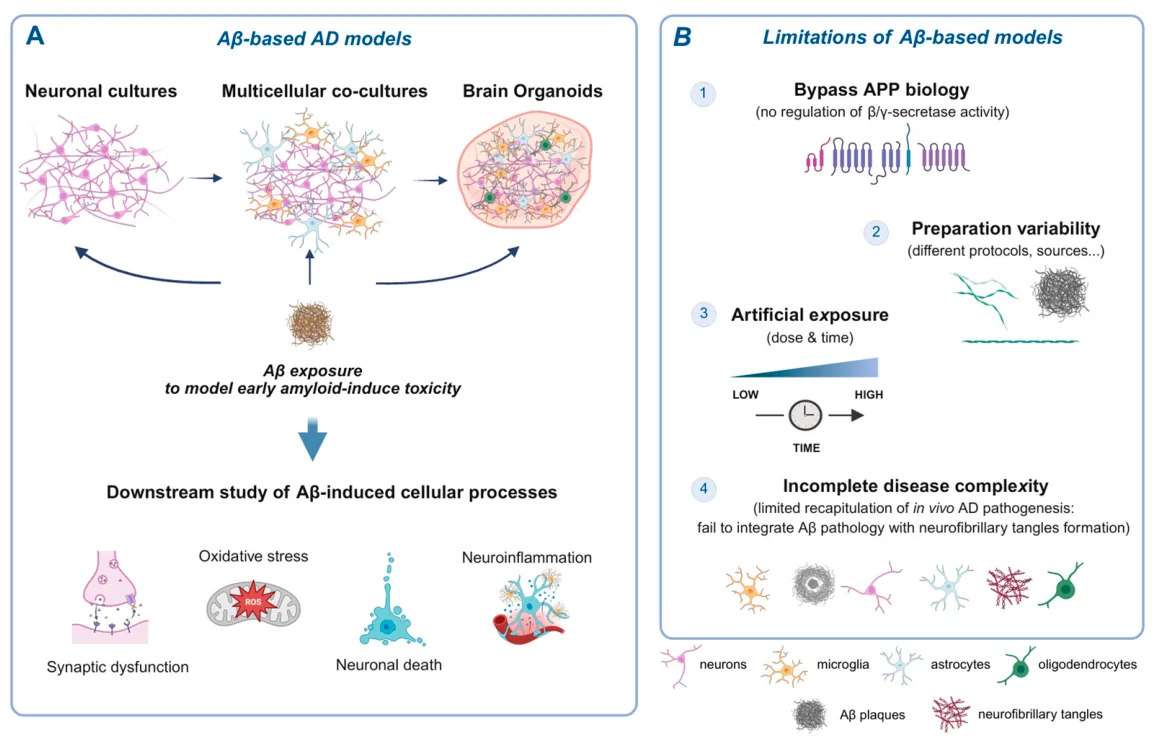
Advantages and limitations of exogenous Aβ exposure in hiPSC-based AD models. (**A**). Schematic overview of Aβ exposure-based models, ranging from neuronal cultures to multicellular co-cultures and brain organoids. These models are best suited for investigating early amyloid-induced toxicity, including synaptic dysfunction, oxidative stress, neuroinflammation, and neuronal death. (**B**). Exogenous Aβ oligomer models reproduce downstream amyloid-induced cellular responses but do not recapitulate endogenous APP processing or the mechanisms regulating Aβ production. Their main limitations include variability in oligomer preparations, dose- and time-dependent effects, and the inability to mimic the chronic, multicellular complexity of AD. This figure was created with BioRender.com.

Overall, approaches employing acute Aβ exposure and genetically driven hiPSC models, while not interchangeable, address complementary issues. Exogenous Aβ administration provides fine control over parameters, such as peptide species, concentration, and exposure time, thus proving especially suitable for dissecting early amyloid-driven toxicity (Figure 2A). Differently, hiPSC models carrying AD-associated mutations or risk variants preserve endogenous APP processing within a defined genetic background and enable the investigation of genetically driven mechanisms, including altered Aβ production, endosomal dysfunction, and cell vulnerability. However, these models require control of differentiation protocols and recapitulate monogenic AD or individual risk pathways rather than the complex heterogeneity of sporadic LOAD.

### 3.3. Limitations and Methodological Challenges of Aβ Exposure Models

Despite their widespread use, exogenous Aβ-based models also present important limitations that should be carefully considered. Primarily, such models fail to integrate amyloid pathology with the progressive development of tau hyperphosphorylation and neurofibrillary tangle formation. In contrast, several genetically engineered models, including familial AD hiPSC-derived neurons carrying APP or PSEN1 mutations and three-dimensional cerebral organoids, develop both amyloid accumulation and tau-related abnormalities over time, thereby more faithfully reproducing the pathological interplay observed in the human disease. Similarly, transgenic animal models frequently exhibit combined amyloid and tau pathology, although the temporal sequence and severity of these features vary according to the specific genetic background [1,61,62,63].

Indeed, the direct application of Aβ peptides bypasses the endogenous amyloidogenic processing of APP, thereby failing to reproduce the dynamic regulation of β- and γ-secretase activity, APP trafficking, endosomal dysfunction, and intracellular Aβ production that occur during AD pathogenesis [41,64]. Consequently, these models are well suited to investigate cellular responses to Aβ exposure such as synaptic dysfunction, oxidative stress, neuroinflammation, and neuronal death, but cannot provide mechanistic insight into the upstream events governing Aβ generation, processing, secretion, and clearance.

Another major challenge concerns the preparation and characterization of soluble Aβ oligomers. Different aggregation protocols, peptide sources, incubation conditions, solvent compositions, and purification methods generate heterogeneous mixtures of Aβ species that differ in size, conformation, stability, and biological activity [40,65,66]. Because Aβ exists as a dynamic equilibrium of monomers, low-molecular-weight oligomers, protofibrils, and fibrils, even minor variations in experimental procedures can substantially alter the composition of the preparation and, consequently, its neurotoxic properties [40]. As a consequence, experimental outcomes may vary considerably among studies, complicating the comparison, reproducibility, and interpretation of published findings [41,65].

Furthermore, both the concentration and duration of Aβ exposure strongly influence the cellular response, ranging from reversible synaptic dysfunction and impaired long-term potentiation at low nanomolar concentrations to oxidative stress, mitochondrial dysfunction, calcium dyshomeostasis, and overt neuronal death at higher concentrations or after prolonged exposure [40,67,68]. Another important limitation is that acute exogenous exposure does not fully recapitulate the chronic and progressive accumulation of Aβ occurring in the human brain, nor does it reproduce the complex interactions among neurons, astrocytes, microglia, vascular cells, and the extracellular matrix that contribute to AD pathogenesis in vivo [41,69]. Nevertheless, despite these methodological limitations, soluble Aβ oligomers remain the most widely accepted experimental tool for reproducing early amyloid-induced synaptic toxicity and are best considered as complementary platforms for defining downstream cellular responses to amyloid stress, particularly those associated with synaptic failure that precedes overt neurodegeneration [40,67,69] (Figure 2B).

Finally, although amyloid deposition is widely considered an initiating event in AD pathogenesis, increasing evidence indicates that tau pathology is the strongest correlate of neuronal loss and cognitive decline, and that both pathological processes synergistically drive disease progression rather than acting independently [70]. A route to disentangling such Aβ–tau interplay from the possibly confounding effects of exogenous exposure protocols may take advantage of isogenic CRISPR-engineered hiPSC lines, which allow dissection of the causal contribution of defined genetic variants, in a controlled human genetic background.

## 4. Modeling Genetic Causality Using CRISPR-Engineered Isogenic hiPSC

The introduction of hiPSC technology combined with CRISPR/Cas9 genome editing has revolutionized the study of several diseases, including AD, by enabling the generation of isogenic cellular systems, in which disease-associated variants are introduced into or corrected within an identical genetic background. Unlike conventional patient-derived hiPSC models, whose interpretation is often complicated by interindividual genetic variability, isogenic pairs differ exclusively at the genetic locus of interest. This strategy has proven to be particularly important, minimizing the confounding effects of interindividual genetic variability, allowing causal genotype–phenotype relationships to be established with greater precision than conventional patient-derived or overexpression models. CRISPR-engineered isogenic models have provided an opportunity to dissect the molecular mechanisms underlying AD, allowing the identification of subtle mutation-dependent phenotypes, while distinguishing primary pathogenic mechanisms from secondary or background genetic effects. This distinction is particularly relevant in AD, where many genetic variants represent risk modifiers rather than direct disease-causing mutations. Consequently, isogenic pairs have become the gold standard for dissecting the contribution of individual AD-associated variants to disease pathogenesis, providing a unique framework to distinguish causal mechanisms from genetic associations [71,72].

### CRISPR-Engineered hiPSCs to Model Causal Mutations and Risk Variants of AD

Familial AD (FAD), although accounting for less than 1% of all AD cases, has provided invaluable mechanistic insights into the molecular mechanisms underlying disease initiation. Highly penetrant mutations in APP, PSEN1, and PSEN2 alter APP processing by increasing the production or aggregation propensity of Aβ, particularly the aggregation-prone Aβ42 peptide, thereby supporting the amyloid cascade hypothesis [73]. CRISPR-engineered hiPSC models carrying these mutations have faithfully reproduced key molecular phenotypes, including elevated Aβ42/Aβ40 ratios, endosomal dysfunction, impaired synaptic activity, altered calcium homeostasis, mitochondrial abnormalities, and increased tau phosphorylation. Importantly, correction of these mutations in isogenic control lines reverses many of these pathological alterations, providing compelling evidence for their direct pathogenic role.

Several studies have established the value of such an approach (Table 1). One of the earliest demonstrations was provided by Paquet et al. (2016), who generated heterozygous and homozygous hiPSC lines carrying the APP Swedish (KM670/671NL) and PSEN1 M146V mutations, showing gene dosage-dependent increases in Aβ42 production and Aβ42/Aβ40 ratios while validating the use of precise CRISPR-mediated engineering for disease modeling [74]. Similarly, correction of the PSEN2 N141I mutation in patient-derived hiPSCs restored both the elevated Aβ42/Aβ40 ratio and the electrophysiological abnormalities observed in basal forebrain cholinergic neurons, establishing a direct causal relationship between the mutation and the neuronal phenotype [75]. Using isogenic hiPSC lines harboring the PSEN1 ΔE9 mutation, Woodruff et al. (2013) demonstrated that this pathogenic variant selectively impairs γ-secretase activity without abolishing other presenilin functions, while also revealing defects in APP trafficking, endosomal recycling, and axonal transport [76]. Finally, Kwart et al. (2019) produced one of the largest CRISPR-engineered panels of isogenic hiPSC neurons carrying multiple APP and PSEN1 mutations [77]. Despite the genetic heterogeneity of these variants, comparative transcriptomic and cellular analyses revealed convergence toward a common phenotype characterized by enlarged Rab5-positive early endosomes and accumulation of APP β-C-terminal fragments (β-CTFs), identifying β-CTFs rather than Aβ as the principal drivers of early endosomal dysfunction. Notably, abnormalities in endosomal trafficking have emerged as one of the earliest and most reproducible phenotypes across CRISPR-engineered APP and PSEN mutant neurons, suggesting that disruption of the endolysosomal system precedes neurodegeneration.

Collectively, these studies demonstrate that CRISPR-engineered isogenic hiPSC models faithfully reproduce the earliest pathogenic consequences of APP and PSEN mutations in human neurons.

Unlike FAD, LOAD results from the combined contribution of multiple genetic variants, each exerting a relatively modest effect on disease susceptibility. In this context, CRISPR/Cas9 genome editing has provided a powerful strategy to directly interrogate the functional contribution of individual LOAD-associated variants while minimizing the confounding effects of genetic background. Among LOAD-associated genes, APOE represents the strongest genetic risk factor for sporadic AD. The first landmark study was reported by Lin et al. (2018), who generated isogenic hiPSC lines by converting APOE3 to APOE4 and vice versa [6]. Differentiation into neurons, astrocytes, and microglia demonstrated that the APOE4 allele promotes cell-type-specific AD phenotypes, including increased Aβ42 secretion, impaired amyloid uptake, dysregulated cholesterol metabolism, enhanced tau phosphorylation, synaptic alterations, and pro-inflammatory transcriptional programs. Importantly, CRISPR-mediated correction of APOE4 to APOE3 largely rescued these pathological phenotypes, establishing APOE4 as a causal driver of LOAD pathology. These findings also highlight the contribution of APOE4 to neuroimmune dysfunction, as alterations in lipid homeostasis and amyloid handling in glial cells may influence inflammatory responses and, consequently, neuronal vulnerability. Subsequently, Wang et al. (2018) generated isogenic hiPSC-derived neurons by correcting the APOE4 allele to APOE3, demonstrating that gene correction normalized tau phosphorylation, restored GABAergic neuronal function, reduced neuronal degeneration, and reversed multiple APOE4-associated toxic phenotypes, providing one of the strongest demonstrations of the value of isogenic genome-edited human models for LOAD [78].

CRISPR/Cas9 editing has also been extensively applied to investigate endosomal trafficking, a cellular pathway increasingly recognized as an early and convergent mechanism in AD pathogenesis. SORL1, a major LOAD risk gene encoding a key regulator of intracellular protein sorting, has been extensively investigated in this context. Knupp et al. (2020) generated SORL1-knockout isogenic hiPSC lines and demonstrated that loss of SORL1 selectively induced enlargement of Rab5-positive early endosomes and defective endosomal trafficking in neurons, independently of amyloidogenic APP processing, whereas microglia were largely unaffected [79]. These findings demonstrated that neuronal endosomal dysfunction represents an early cell-autonomous consequence of SORL1 deficiency.

Similarly, genome editing approaches have revealed the contribution of immune-related risk genes to AD pathogenesis. Using human hiPSC-derived microglia, carrying the AD-associated TREM2 R47H variant, Garcia-Reitboeck et al. (2018) demonstrated impaired phagocytosis and reduced microglial functionality [80]. These findings were further extended by Piers et al. (2020), who found that CRISPR-engineered TREM2 R47H- and TREM2-knockout hiPSC-derived microglia show defective immunometabolic reprogramming, reduced mitochondrial respiratory capacity, impaired glycolytic switching, and diminished inflammatory responses [81]. Together, these findings highlight a critical role of TREM2 in maintaining microglial homeostasis and suggest that impaired immune–metabolic responses contribute directly to AD pathogenesis.

Beyond APOE4, TREM2, and SORL1, recent advances in CRISPR/Cas9 technologies have enabled the systematic investigation of additional GWAS-identified AD risk loci, shifting the field from the study of individual genes toward the identification of convergent disease-associated molecular networks including PLCG2, CD33, ABCA7, BIN1, and PICALM.

ABCA7, one of the strongest LOAD risk genes, identified by genome-wide association studies, has been investigated using CRISPR/Cas9-engineered hiPSC models. Guan et al. (2023) generated a homozygous ABCA7-knockout hiPSC line through CRISPR/Cas9-mediated genome editing, providing a genetically defined platform to study the contribution of ABCA7 loss of function to AD-related phenotypes [82]. Kawatani et al. (2024) demonstrated that ABCA7 deficiency in hiPSC cortical neurons and cerebral organoids disrupts mitochondrial lipid metabolism, reducing mitochondria-associated phospholipids such as cardiolipin and phosphatidylglycerol, impairing mitochondrial respiration, increasing oxidative stress, and compromising neuronal network activity [83]. These findings provide direct evidence that ABCA7 loss-of-function variants contribute to AD susceptibility through mitochondrial and synaptic dysfunction.

Other major LOAD-associated genes, including BIN1 and PICALM, have been targeted with CRISPR-based approaches to interrogate their functional contribution in human cellular models. Both genes encode regulators of clathrin-mediated endocytosis, a process essential for membrane dynamics, intracellular transport, and APP trafficking. CRISPR-mediated gene perturbation was employed in human brain organoids to demonstrate that reduced expression of AD risk genes, including BIN1 and PICALM, alters transcriptional programs related to endocytosis and cholesterol metabolism, highlighting their role in cell responses to amyloid pathology [84,86,87,88].

Finally, MAPT, encoding the microtubule-associated protein tau, has been studied using CRISPR/Cas9 knockout hiPSC-derived neuronal models to dissect the contribution of specific tau isoforms to AD-related neuronal vulnerability. Buchholz et al. (2025) generated MAPT-knockout hiPSCs differentiated into glutamatergic neurons and demonstrated that tau depletion modifies neuronal morphology and protects against Aβ oligomer-induced dysfunction [85]. Conversely, re-expression of the 1N4R tau isoform restores susceptibility to amyloid toxicity and promotes pathological tau phosphorylation, identifying tau isoform-specific mechanisms underlying Aβ–tau interaction in AD.

Despite the genetic diversity of the variants investigated, several phenotypic patterns emerge across CRISPR-engineered AD models. Alterations in endosomal trafficking represent one of the most consistent phenotypes, particularly in APP, PSEN1, and SORL1 models, although the underlying mechanisms differ. APP and PSEN1 mutations can produce early endosomal abnormalities associated with altered APP processing, whereas SORL1 deficiency directly disrupts neuronal endosomal trafficking independently of amyloidogenic APP processing. Disturbances in lipid and mitochondrial homeostasis also emerge across genetically distinct models, although their cellular manifestations differ. APOE4 produces broad, cell-type-specific alterations involving cholesterol and lipid metabolism, synaptic function, and microglial responses, whereas ABCA7 deficiency predominantly affects mitochondrial lipid composition, oxidative metabolism, and neuronal network function. Immune-related variants such as TREM2 further highlight the contribution of microglial dysfunction, affecting phagocytosis, survival, lipid handling, and immunometabolic adaptation. In contrast, MAPT perturbation highlights a more specific contribution of tau to neuronal vulnerability, as tau depletion can modify neuronal responses to Aβ oligomers. Thus, rather than producing identical phenotypes, distinct AD-associated variants appear to converge on partially overlapping cellular pathways while retaining substantial gene-, variant-, and cell-type-specific effects.

Overall, the application of CRISPR/Cas9-based genome editing to both FAD mutations and LOAD risk variants has progressively expanded the utility of hiPSC models, moving from the investigation of individual disease genes to the identification of convergent pathogenic pathways. By enabling the generation of isogenic human cellular systems, these approaches have provided unprecedented insights into how distinct genetic alterations converge on shared pathogenic pathways, including amyloid processing, endosomal trafficking, lipid dysregulation, mitochondrial dysfunction, neuroinflammation, and tau-mediated neuronal vulnerability. This shift from gene-specific characterization toward the identification of shared molecular mechanisms represents a major advance in understanding AD pathogenesis (Table 1). These models not only provide a robust platform for elucidating the functional consequences of individual genetic variants but also enable high-throughput drug screening and mechanistic studies, thereby bridging the gap between human genetics and translational neuroscience [71,89].

While neuronal cultures and genetically engineered hiPSC-derived neurons have provided fundamental insights into AD pathogenesis, conventional two-dimensional (2D) cell culture models remain limited in their ability to recapitulate the complexity of neurodegenerative diseases. Indeed, although neuronal monolayer cultures have greatly advanced our understanding of cell-autonomous mechanisms underlying AD, they fail to reproduce the structural complexity and multicellular interactions that characterize the human brain [90,91]. Cells grown on flat surfaces fail to reproduce the three-dimensional (3D) architecture and regional organization of the brain, limiting the study of selective neuronal vulnerability. Moreover, these models often consist of a single cell type, lacking intricate interactions among neurons, astrocytes, microglia, oligodendrocytes, and endothelial cells, thereby limiting the study of neuroinflammatory responses, metabolic regulation, and neurovascular interactions [90,91]. They also fail to mimic the neurovascular unit, preventing investigation of blood–brain barrier function, metabolic support, and protein clearance mechanisms [92,93]. Last but not least, 2D cultures cannot accurately model the spatial propagation of pathological protein aggregates, a key feature driving disease progression in major neurodegenerative disorders [90,91,94].

Based on the above considerations, distinct from genetically engineered models, which reproduce pathogenic molecular cascades in a controlled, reductionist system, the anatomical and functional complexity of the susceptible brain regions has been addressed by developing tissue-like platforms, namely brain organoids.

## 5. Beyond Neurons: Brain Organoids and Multicellular Neuroglial Systems

The development of hiPSC-derived brain organoids has marked a major conceptual shift from reductionist neuronal systems toward tissue-level models that more faithfully recapitulate human cortical architecture, cell-type heterogeneity, and intercellular communication. By allowing neural cells to self-organize within a 3D-structured environment, organoids enable the investigation of disease mechanisms at a level that extends beyond the cell-autonomous phenotype (Table 2).

Brain organoids contain diverse neural populations, including neural progenitor cells, excitatory and inhibitory neurons, astrocytes, and, upon prolonged maturation or through dedicated differentiation protocols, oligodendrocytes. This cellular complexity recreates a microenvironment in which disease-associated signaling emerges from coordinated cellular interactions rather than isolated neuronal dysfunction [90,91,96].

The transition to 3D multicellular systems has enabled the spontaneous development of hallmark AD pathological features that are difficult or impossible to reproduce in conventional monolayer cultures. Brain organoids derived from patients carrying FAD mutations in APP, PSEN1, or PSEN2 exhibit key disease-related phenotypes, including progressive extracellular amyloid-β deposition, increased Aβ42/Aβ40 ratios, tau hyperphosphorylation, endosomal abnormalities, synaptic dysfunction, oxidative stress, and neuronal degeneration, closely resembling early stages of human disease [63,95].

Likewise, organoids generated from patients carrying the APOE4 genotype or other sporadic AD risk variants reproduce impaired cholesterol metabolism, altered neuronal maturation, astrocytic dysfunction, and enhanced neuroinflammatory responses, highlighting the ability of these models to capture both familial and sporadic pathogenic mechanisms within a human genetic background [96]. Furthermore, long-term organoid cultures allow pathological processes to evolve over time, providing a unique opportunity to investigate the temporal relationships between amyloid accumulation, tau pathology, gliosis, and neuronal vulnerability [96,103].

### 5.1. Modeling Neuron–Microglia Interactions in Brain Organoids

One of the most significant advances in recent years has been the incorporation of microglia into brain organoid platforms, enabling the investigation of neuroimmune interactions in a human context. Although most guided cerebral organoids lack bona fide mesoderm-derived microglia, spontaneous microglia-like populations have been reported in unguided organoid protocols. Nevertheless, the majority of current AD models rely on the deliberate incorporation of hiPSC-derived microglia through co-culture or assembloid approaches to achieve reproducible neuroimmune interactions [97,98]. These models have demonstrated that microglia regulate neuronal maturation, synaptic pruning, circuit refinement, clearance of apoptotic debris, and inflammatory signaling while dynamically responding to amyloid pathology. In an AD context, microglia surrounding amyloid deposits acquire disease-associated transcriptional signatures, modulate phagocytic activity and release cytokines, chemokines and complement components that influence neuronal survival and function through bidirectional communication with neurons and astrocytes [99,100]. Such neuron–microglia organoid models have proven particularly valuable in investigating the functional consequences of AD risk genes predominantly expressed in immune cells, including TREM2, CD33, PLCG2, APOE and ABI3, which cannot be adequately modeled in neuron-only cultures. Consequently, next-generation organoid and neuroglial co-culture systems are progressively shifting AD research from a neuron-centric perspective into an integrated tissue-level framework enabling a more comprehensive understanding of how genetic susceptibility, amyloid pathology, and neuroinflammation interact during disease progression.

### 5.2. Modeling Astrocyte-Mediated Mechanisms in Brain Organoids

Beyond neurons and microglia, incorporating astrocytes into hiPSC-derived brain organoid and assembloid systems represents a further step toward recapitulating the cellular complexity of the human AD brain [104]. Astrocytes are essential regulators of neuronal homeostasis, contributing to synaptic formation and maintenance, neurotransmitter recycling, calcium signaling, metabolic support, extracellular ion regulation, and maintenance of the blood–brain barrier [105]. In AD, however, astrocytes undergo profound molecular and functional alterations, transitioning from a homeostatic state toward disease-associated phenotypes characterized by altered inflammatory responses, impaired metabolic support, and defective regulation of amyloid and lipid homeostasis [45,106,107].

In the human brain, astrocytes represent the major cellular source of APOE, a key regulator of lipid transport, cholesterol homeostasis, and neuronal metabolic support. The APOE4 isoform alters astrocyte biology by promoting lipid dysregulation, impaired cholesterol trafficking, abnormal lipid droplet accumulation, and reduced capacity to maintain neuronal homeostasis. Therefore, APOE4-associated AD risk is increasingly recognized as a multicellular process in which astrocytic dysfunction contributes to neuronal vulnerability and disease progression [64,108].

Consistent with this concept, APOE4 human hiPSC-derived cerebral organoid models have reproduced several AD-associated phenotypes, including increased amyloid accumulation, synaptic dysfunction, tau pathology, and neuronal degeneration. Importantly, transcriptomic and cellular analyses of APOE4 organoids have revealed alterations in lipid metabolism, cellular stress responses, and glial-associated pathways, suggesting that APOE4 influences disease mechanisms through coordinated effects across different brain cell populations rather than through neuron-autonomous mechanisms alone [6,64].

More recently, chimeric cerebral organoid approaches have enabled the specific contribution of astrocytic versus neuronal APOE4 to be dissected. By generating organoids containing astrocytes and neurons with different APOE genotypes, Huang et al. (2022) demonstrated that astrocytic APOE4 alone was sufficient to promote lipid droplet formation and cholesterol accumulation in neurons [101], whereas the combination of APOE4 expression in both astrocytes and neurons produced the strongest pathological effects.

**Figure 3:**
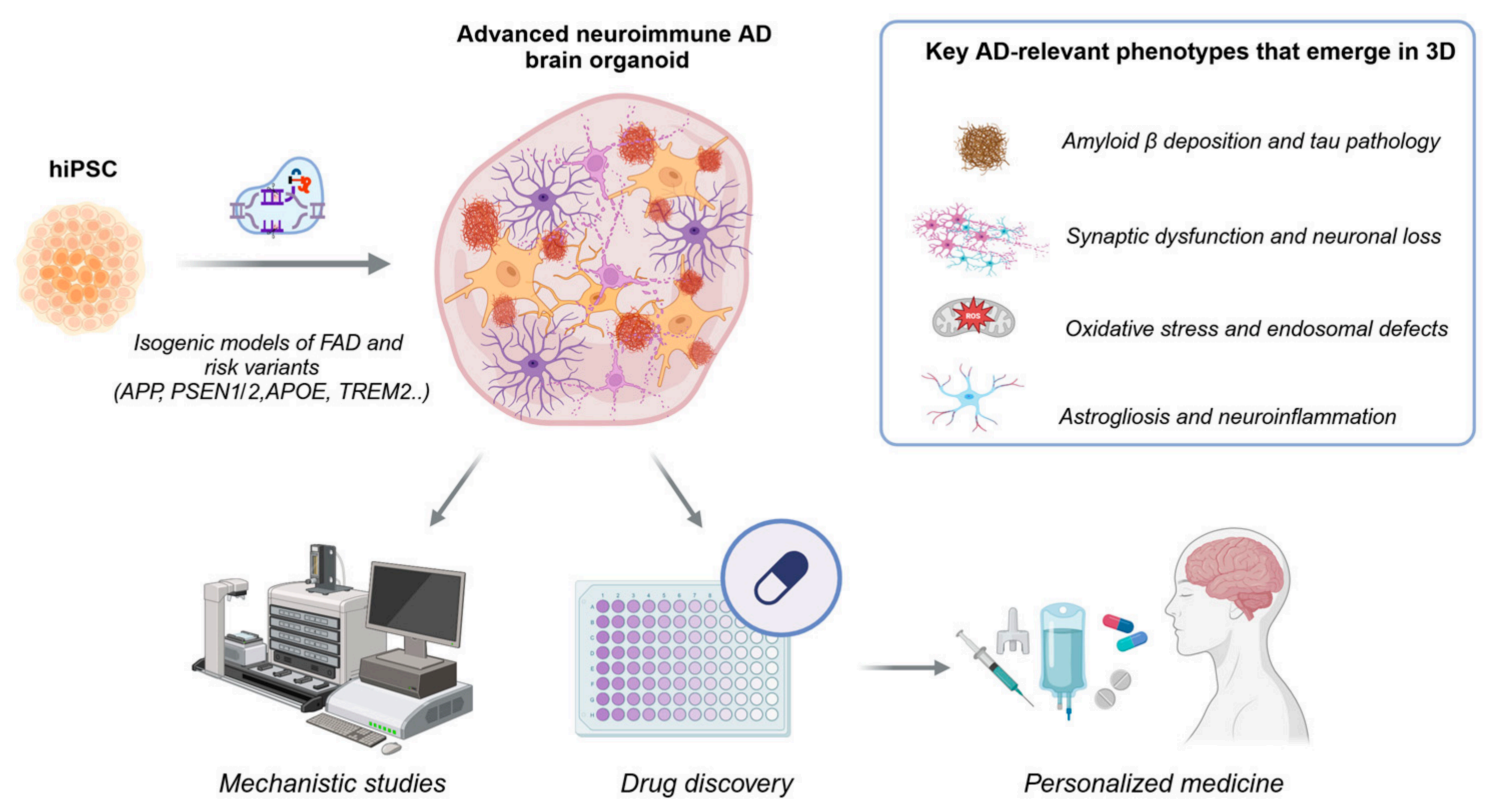
Multicellular neuroimmune brain organoids as next-generation models of AD. Isogenic CRISPR-engineered hiPSCs to model FAD or LOAD are used to generate brain organoids integrating neurons, astrocytes and microglia, enabling the emergence of hallmark AD phenotypes, including amyloid and tau pathology, oxidative stress, endosomal defects, neuroinflammation and neuronal dysfunction/loss. These human 3D systems support disease modeling, target identification and preclinical therapeutic testing. This figure was created with BioRender.com.

These findings highlight the unique value of multicellular organoid systems for resolving cell-type-specific mechanisms of AD risk genes (Figure 3).

### 5.3. Towards Standardization to Improve the Translational Potential of Brain Organoids

Despite their considerable potential, cerebral organoids still present important limitations that constrain their application as reliable models of neurodegenerative diseases. One of the major challenges is their relatively immature developmental state. Most cerebral organoids resemble the transcriptional and functional characteristics of the fetal human brain, making it challenging to model late-onset neurodegenerative processes such as sporadic AD without additional aging-inducing strategies or prolonged culture periods [90,104,109]. Another important limitation is the substantial variability associated with organoid generation, including differences in size, morphology, cellular composition, and developmental trajectories. Such heterogeneity, influenced by differentiation protocols, culture conditions, and maturation time, can complicate the reproducibility and interpretation of disease-associated phenotypes [91,104]. Although this variability has historically limited the standardization of organoid-based experiments, significant methodological improvements have been achieved in recent years. The implementation of optimized differentiation protocols, together with advances in bioreactor technology and culture engineering, has markedly increased organoid reproducibility, yielding highly consistent cellular composition and developmental patterns across independent cultures [110]. For example, single-cell RNA-sequencing analysis of 166,242 cells from 21 individual dorsally patterned forebrain organoids showed that 95% generated a virtually indistinguishable repertoire of cortical cell types and followed similar developmental trajectories [109].

Notably, the residual variability observed between individual organoids may partially reflect the intrinsic biological heterogeneity found among human brains, suggesting that, rather than representing exclusively a technical limitation, organoid-to-organoid differences may also capture physiologically relevant aspects of human brain development. Nevertheless, structural variability, particularly in the size and organization of ventricular-like progenitor zones, remains an important source of heterogeneity that can influence lineage specification and the relative abundance of neuronal and glial populations. Continued refinement of differentiation strategies, quality-control pipelines, and bioengineering approaches will therefore be essential to further improve the robustness and translational value of cerebral organoid models [109,110].

Future advances integrating vascularization, immune components, aging strategies, bioengineered scaffolds, and standardized quality-control pipelines are expected to further enhance the physiological relevance and translational potential of brain organoids for AD research.

## 6. hiPSC-Derived Cells as a Bridge to Regenerative Medicine

Beyond their established role in disease modeling and drug screening, hiPSCs also represent a potential source of cells for regenerative therapy. Patient-derived hiPSCs can be genetically corrected, expanded, and differentiated into disease-relevant cell types before being transplanted back into the donor. In principle, this autologous approach may reduce the risk of immune rejection and limit the need for long-term immunosuppression, while enabling the replacement or functional support of damaged tissues [111,112]. The clinical feasibility of this strategy was first demonstrated in regenerative medicine through the transplantation of autologous hiPSC-derived retinal pigment epithelial cells in a patient with age-related macular degeneration [113]. These early applications provided proof of principle that hiPSC-derived products can be manufactured for patient-specific transplantation, although their broader therapeutic use still depends on demonstrating genomic stability, cellular purity, functional integration, and long-term safety.

Notably, the therapeutic potential of hiPSC-derived neural cells depends not only on their survival after transplantation, but also on their ability to integrate into mature human neural networks. Supporting this possibility, long-term self-renewing neuroepithelial-like stem cells were grafted onto organotypic slices of adult human neocortex obtained from the middle temporal gyrus of patients undergoing epilepsy surgery. Following transplantation, the cells differentiated into layer-associated neuronal populations and established functional afferent and efferent synaptic connections with resident cortical neurons. By showing structural and electrophysiological integration within adult human tissue, this study provides important proof that hiPSC-derived neural progenitors may contribute to the reconstruction or reinforcement of neuronal circuits disrupted by neurodegeneration [114]. Nevertheless, these findings represent a translational bridge rather than evidence of clinical efficacy, as long-term survival, safety, and functional benefit remain to be established in vivo.

### 6.1. Stem Cell-Based Strategies in AD

Conventional pharmacological management of AD primarily relies on symptomatic treatments that alleviate cognitive decline and, to a lesser extent, behavioral and psychological symptoms. Acetylcholinesterase inhibitors, including donepezil, galantamine, and rivastigmine, partially compensate for the loss of cholinergic neurotransmission, whereas memantine, a non-competitive antagonist of N-methyl-D-aspartate receptors (NMDA), limits excitotoxicity [115,116]. However, their clinical benefits are generally modest and transient, as they primarily address downstream functional consequences of neurodegeneration, rather than replacing lost neurons or repairing neural networks. Effectiveness is further constrained by challenges in achieving adequate drug concentrations in the brain, due to the restrictive properties of the blood–brain barrier [117]. Collectively, these limitations emphasize the need for therapeutic strategies not only targeting disease-associated pathways, but also protecting vulnerable cells, thus restoring tissue homeostasis.

Importantly, stem cell-based approaches may provide dual therapeutic benefit by replacing lost or dysfunctional cells and by modifying the pathological brain environment through trophic, immunomodulatory, and neurovascular mechanisms.

Mesenchymal stromal cells (MSCs) were among the first stem cell-based approaches to undergo clinical evaluation in AD. MSCs can be isolated from various sources, including bone marrow, adipose tissue, and perinatal tissues, such as umbilical cord blood. Their therapeutic effects are thought to result primarily from the release of trophic, immunomodulatory, and neuroprotective factors, rather than differentiation into neurons. Through these paracrine mechanisms, MSCs may modulate microglial activation, facilitate amyloid-β clearance, support endogenous neurogenesis, and enrich brain microenvironment [118,119]. This biological rationale formed the basis for initial clinical studies assessing the feasibility and safety of MSC administration in patients with AD.

An open-label phase I trial evaluated the stereotactic administration of human umbilical cord blood-derived MSCs in patients with mild-to-moderate AD. The cells were injected bilaterally into the hippocampus and unilaterally into the right precuneus, two regions significantly affected by Alzheimer’s pathology. The procedure was technically feasible and generally well tolerated. However, due to the small sample size and the absence of a placebo-treated control group, the authors could not determine whether MSC administration resulted in significant changes in disease progression [120].

Whereas MSC-based strategies mainly aim to modify the pathological environment through paracrine mechanisms, hiPSC-derived neural cells offer the distinct possibility of replacing vulnerable cell populations and reconstructing damaged neuronal circuits. This concept was recently explored by Zhang et al. (2026), who generated post-mitotic pallial inhibitory neurons, originating from the medial ganglionic eminence, from human hiPSCs and transplanted them bilaterally into the hippocampus of 10-month-old 5xFAD mice [121]. The transplanted cells differentiated into GABAergic interneuron subtypes and became functionally integrated into the host’s hippocampal circuitry. This integration restored local inhibitory signaling, corrected the disrupted excitatory–inhibitory balance, reduced epileptiform activity, and improved synaptic plasticity and cognitive performance. Notably, these functional benefits occurred without a detectable reduction in amyloid-β levels, suggesting that cell transplantation may improve brain function by repairing circuit-level abnormalities rather than by directly removing the underlying protein aggregates. The use of lineage-defined, post-mitotic cells may also reduce the proliferative and tumorigenic risks associated with less differentiated neural progenitors. However, these findings remain preclinical, and their translation will require confirmation of long-term graft survival, safety, immune compatibility, and functional integration in the considerably more complex environment of the human AD brain.

Although transplantation of defined neuronal populations may restore specific components of damaged neural circuits, the extensive and multicellular pathology of AD raises concerns regarding the sufficiency of replacing a single cell type. Recent advances in three-dimensional culture systems have generated interest in human hiPSC-derived brain organoids as more sophisticated grafting platforms. In contrast to dissociated cell preparations, brain organoids are self-organizing neural tissues in which progenitor cells, differentiating neurons, and glial populations develop within a partially organized three-dimensional architecture [122,123].

Cortical organoids typically feature ventricular-like cavities surrounded by apical radial glial cells, as well as intermediate and outer radial glial progenitors that generate neurons migrating toward cortical plate-like regions [122]. With extended differentiation, these structures can produce layer-associated excitatory neurons, astrocytes, and functional synaptic networks [123]. Depending on protocols, additional cellular components, such as inhibitory interneurons, oligodendroglial cells, microglia, and vascular-like cells, may be incorporated or combined using assembloid approaches. Nevertheless, cortical organoids do not recapitulate the full cellular and anatomical complexity of the adult human brain and generally lack intrinsic functional vasculature and a complete immune compartment [84,97,124,125].

The partially preserved cytoarchitecture may provide advantages over isolated cell transplantation by offering a preassembled neural niche that facilitates cell–cell interactions and coordinated neuronal and glial network development. Supporting this possibility, intact cerebral organoids showed greater long-term survival and tissue regeneration than dissociated cells following transplantation into the mouse cortex [126]. Indeed, host-derived blood vessels and microglia infiltrate the graft, enhancing cell survival and promoting further differentiation. Grafted organoids have demonstrated the ability to extend long-range axonal projections and form both afferent and efferent connections with the surrounding brain tissue [127,128]. Notably, cortical organoids implanted into the developing rat cortex received corticocortical and thalamocortical inputs, responded to sensory stimulation, and generated outputs that influenced the behavior of the host [129].

These properties expand the concept of cell replacement to include the implantation of organized, multicellular neural tissue. However, brain organoid transplantation remains highly experimental and has not yet been established as a therapeutic strategy for AD. Whether grafted organoids can survive and mature within the chronically inflammatory, amyloid- and tau-rich environment characteristic of the AD brain has not been directly tested. Nevertheless, exposure of vascularized neuroimmune organoids to human AD brain extracts was sufficient to induce amyloid- and tau-like pathology, neuroinflammation, increased microglial synaptic pruning, synaptic and neuronal loss, and impaired network activity. These findings support the concern that donor-derived tissue could remain vulnerable to pathological factors originating from the host brain, although this possibility still requires direct evaluation in transplantation models [102].

Thus, whereas transplantation of lineage-defined neurons seeks to restore specific circuit components, organoid-based approaches represent a more ambitious effort to reconstruct a multicellular tissue environment, the safety and therapeutic relevance of which have yet to be demonstrated in AD.

### 6.2. Stem Modeling Neuronal Aging in hiPSC-Derived Systems: A Major Challenge for AD Research

A major limitation of hiPSC-based AD models is their limited ability to recapitulate neuronal aging, the strongest risk factor for disease onset. Reprogramming resets many age-associated molecular signatures, including DNA methylation patterns, mitochondrial alterations, telomere shortening, and senescence-related transcriptional programs. Therefore, hiPSC-derived neurons typically retain immature, fetal-like features and may therefore underestimate age-dependent mechanisms of LOAD, including mitochondrial dysfunction, impaired proteostasis, lysosomal defects, and genomic instability [130,131].

Several strategies have been developed to overcome this limitation, including prolonged neuronal maturation that promotes the gradual acquisition of adult neuronal features, including increased synaptic complexity, enhanced electrophysiological activity, and metabolic maturation, although this partially reproduces the multifactorial nature of human brain aging.

A complementary method consists of experimentally inducing aging through the expression of progerin, a truncated form of lamin A associated with premature aging [130,132].

Additional strategies use oxidative stress, mitochondrial impairment, inflammatory stimuli, or metabolic challenges to reproduce selected features of aged neurons, thereby improving the detection of AD-associated phenotypes in otherwise immature neuronal cultures.

An alternative approach involves the direct conversion of somatic cells into induced neurons (iNs), bypassing the pluripotent state. Direct conversion largely preserves donor age-associated epigenetic and transcriptomic signatures, providing complementary models for investigating late-onset neurodegenerative disorders [130].

Although each approach reproduces only selected aspects of neuronal aging, their integration with CRISPR-engineered isogenic models, brain organoids, and single-cell multi-omics may provide more physiologically relevant platforms for investigating the interaction between genetic susceptibility and aging in sporadic AD.

## 7. Conclusions and Future Perspectives

**Figure 4:**
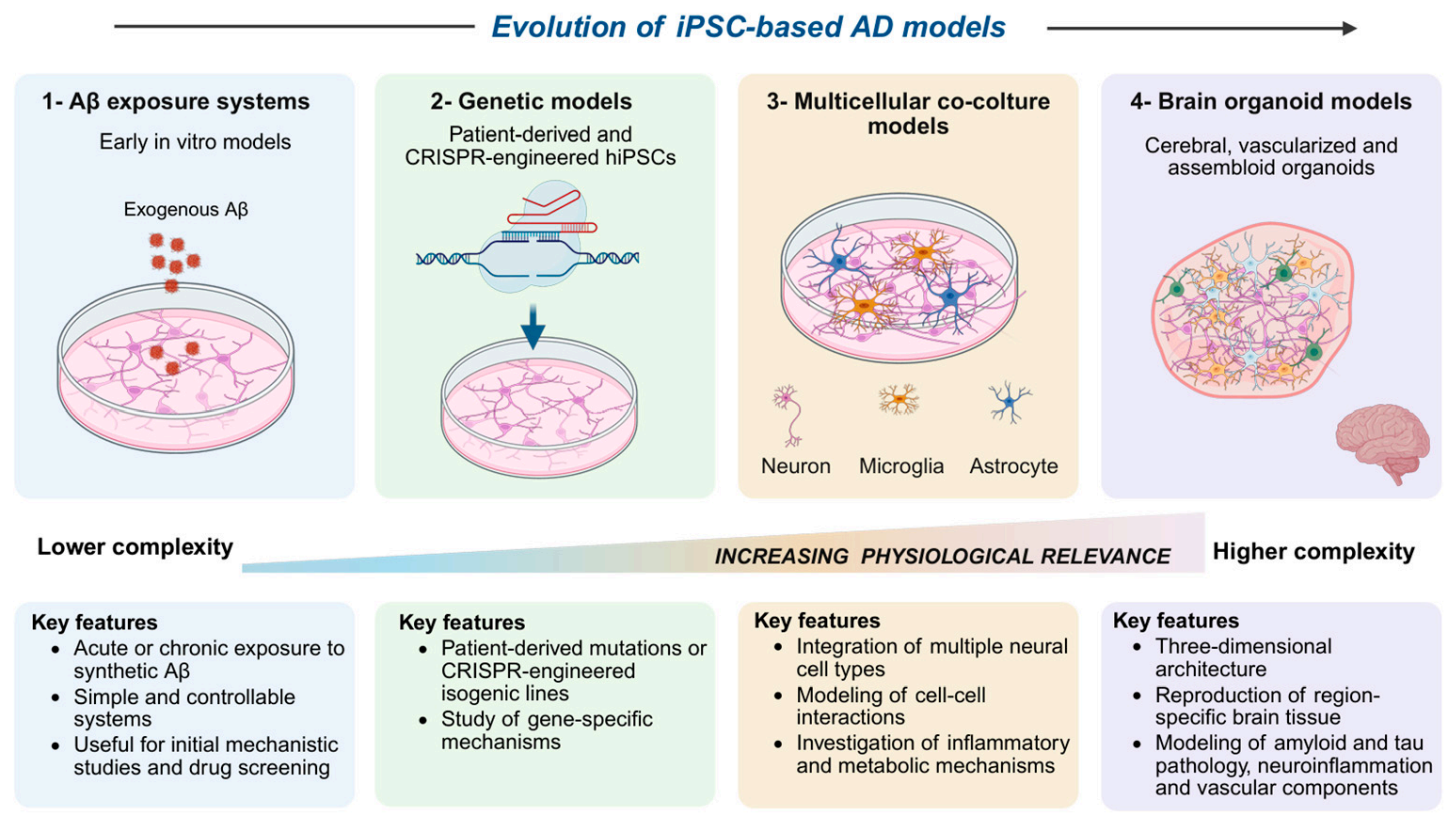
Evolution of hiPSC-based models of Alzheimer’s disease. Schematic overview of the progression from conventional in vitro systems based on exogenous amyloid-β (Aβ) exposure to patient-derived and CRISPR-engineered hiPSC models, multicellular co-culture systems, and three-dimensional brain organoids. Each approach provides complementary insights into AD pathogenesis, ranging from amyloid-associated neurotoxicity and gene-specific mechanisms to cell–cell interactions and tissue-level pathological processes.

Human iPSC-based models have profoundly transformed AD research by providing experimental systems that combine human genetic background with unprecedented cellular versatility. The progressive transition from acute neurotoxicity models to CRISPR-engineered isogenic lines and multicellular brain organoids has enabled increasingly accurate dissection of the molecular and cellular mechanisms underlying both familial and sporadic AD (Figure 4). Rather than representing competing approaches, these systems should be considered as complementary, each mimicking distinct aspects of disease pathogenesis and together providing a comprehensive view of AD biology.

Despite these advances, several limitations continue to constrain the ability of hiPSC-based systems to reproduce the complexity of AD, particularly its late-onset and sporadic forms. Future technological developments should therefore address three interconnected challenges: the limited representation of neuronal aging, the incomplete cellular and structural complexity of current models, and the variability that affects their reproducibility and translational relevance.

A central challenge is the incorporation of age-associated features into human neuronal models. Prolonged maturation, experimentally induced aging, and iNs offer complementary strategies to investigate age-dependent disease mechanisms, although each capture only selected features of biological aging.

A second important direction is increasing the physiological complexity of human models through the incorporation of astrocytes, microglia, and vascular components into three-dimensional systems. Such approaches may improve the investigation of neuroimmune interactions and cell-type-specific vulnerability.

Finally, improving reproducibility and comparability across experimental platforms will be essential for translating mechanistic findings into reliable therapeutic insights. Standardized differentiation protocols, quantitative quality-control criteria, and single-cell multi-omics will be crucial for improving reproducibility and distinguishing disease-associated phenotypes from technical and cellular variability.

Beyond disease modeling for research, hiPSC-derived systems are also opening new opportunities for precision medicine, target validation, and regenerative approaches. The therapeutic potential of hiPSC-derived cell replacement is currently being explored in several neurodegenerative diseases, although its feasibility largely depends on the anatomical distribution and subset specificity of neuronal loss. In Huntington’s disease, the transplantation of neural progenitors derived from hiPSCs with the suppression of the mutant HTT gene aims to restore vulnerable striatal GABAergic neurons, whereas in amyotrophic lateral sclerosis, the observed benefits in animal models mainly derive from neurotrophic and anti-inflammatory effects, rather than extensive replacement of motor neurons [133,134]. Parkinson’s disease currently represents the most advanced clinical application, owing to the relatively selective degeneration of midbrain dopaminergic neurons. A recent phase I/II clinical trial demonstrated the feasibility and safety of transplanting allogeneic hiPSC-derived dopaminergic progenitors, providing encouraging evidence of graft survival and preliminary functional improvements [135]. Although far more complex, as it involves widespread neuronal loss together with glial and vascular dysfunction, this trial provides important proof of principle for the clinical translation of hiPSC-based therapies and offers valuable insights into graft production, immune compatibility, and long-term safety.

Overall, human stem cell-based technologies are progressively bridging the gap between human genetics, disease mechanisms, and translational neuroscience. Continued advances in genome engineering, multicellular modeling, and human neuron-based platforms will likely accelerate the identification of disease mechanisms, therapeutic targets, and ultimately disease-modifying interventions for AD.

## Figures and Tables

**Table 1:** Summary of major CRISPR/Cas9- engineered hiPSC models used to investigate AD-associated genetic variants. Adapted from findings reported by [6,74,75,76,77,78,79,80,81,82,83,84,85].

CRISPR-Edited hiPSC Lines	Cellular Phenotypes	Pathological Phenotype	Refs.
APP (Swedish KM670/671NL, London V717I)	↑Aβ42/Aβ40, synaptic defects, ER stress, enlarged Rab5-positive endosomes	Amyloid dysregulation and early endosomal dysfunction	[74]
PSEN1 (M146L, A246E, L150P, ΔE9)	↑Aβ42/Aβ40, γ-secretase defects, ↑pTau, impaired endosomal recycling, defective lysosomal function and axonal transport	Altered amyloid processing and endolysosomal dysfunction	[76,77]
PSEN2 (N141I KI/correction)	↑Aβ42/Aβ40 Ca2+ dysregulation	Amyloid dysregulation and neuronal functional abnormalities	[75]
APOE (ε4-ε3 conversion)	↑Aβ, ↑pTau, lipid dysregulation, synaptic alterations, astrocyte dysfunction, impaired cholesterol transport ↑pro-inflammatory transcriptional programs	Lipid dysregulation and amyloid dysfunction	[6,78]
SORL1 (KO/missense)	↑Aβ ↑pTau Defective endosomal trafficking	Endosomal trafficking defects independent in APP processing	[79]
TREM2 (R47H, KO)	↓ immunometabolic reprogramming ↓ mitochondrial respiratory capacity ↓ inflammatory responses Defective phagocytosis	Impaired microglial phagocytosis and microglia functionality	[80,81]
ABCA7 (KO)	Mitochondrial and lipid metabolism defects, oxidative stress and neuronal network dysfunction	Altered mitochondrial and lipid metabolism	[82,83]
BIN1 (KO)	Endosomal and cholesterol metabolism defects, synaptic dysfunction	Lipid dysregulation and neuronal dysfunction	[84,86]
PICALM (KO)	Endocytosis and cholesterol metabolism defects	Endocytic and cholesterol metabolism defects	[84,87]
MAPT (KO)	↓ tau toxicity	Protection against Aβ oligomer-induced dysfunction	[85]

**Table 2:** Comparative overview of hiPSC-derived brain organoid models used to investigate Alzheimer’s disease.

Organoid Model	Experimental Approach	Main AD-Related Findings	Representative Studies
FAD-derived cerebral organoids	Organoids carrying APP, PSEN1 or PSEN2 mutations	Amyloid accumulation, altered Aβ42/Aβ40 ratio, tau phosphorylation and neuronal dysfunction	[63,95]
APOE4 cerebral organoids	Organoids carrying AD-associated APOE genotypes	Impaired lipid metabolism, altered neuronal maturation, astrocytic dysfunction, enhanced neuroinflammatory responses, glial-associated alterations	[6,64,96]
Microglia-containing organoids	Incorporation of hiPSC-derived microglia or microglia-like populations	Amyloid-associated microglial responses, phagocytosis, inflammatory signaling and synaptic pruning	[97,98,99,100]
Chimeric neuroglial organoids	Combination of neurons and astrocytes with distinct APOE genotypes	Identification of astrocyte-dependent effects of APOE4 on neuronal cholesterol accumulation and lipid droplets	[101]
“Grafted” organoids	Integration of vascularized neuroimmune organoids and human AD brain extracts	Modeling of amyloid- and tau-associated pathology, neuroinflammation and neuronal network dysfunction	[102]

## Data Availability

No new data were created or analyzed in this study.

## Abbreviations

The following abbreviations are used in this manuscript:

Aβ	Amyloid-β peptide
β-CTFs	Amyloid precursor protein β-C-terminal fragments
ABCA7	ATP-binding cassette subfamily A member 7
AD	Alzheimer’s disease
APOE	Apolipoprotein E
APP	Amyloid precursor protein
BIN1	Bridging integrator 1
CD33	Cluster of differentiation 33
CLU	Clusterin
CR1	Complement C3b/C4b receptor 1
CRISPR/Cas9	Clustered regularly interspaced short palindromic repeats/CRISPR-associated protein 9
EOAD	Early-onset Alzheimer’s disease
FAD	Familial Alzheimer’s disease
GABA	γ-Aminobutyric acid
GWAS	Genome-wide association study/studies
hiPSC	Human induced pluripotent stem cell
HTT	Huntingtin
iNs	Induced neurons
KI	Knock-in
KO	Knockout
LOAD	Late-onset Alzheimer’s disease
MAPT	Microtubule-associated protein tau
MSC	Mesenchymal stromal cell
